# Animal Species Classification from Vocalizations Using Cochlear-Inspired Audio Features and Machine Learning

**DOI:** 10.3390/biomimetics10120830

**Published:** 2025-12-11

**Authors:** Karim Youssef, Julien Moussa H. Barakat, Ghina El Mir, Sherif Said, Samer Al Kork, Alaa Eleyan

**Affiliations:** 1 College of Engineering and Technology, American University of the Middle East, Egaila 54200, Kuwait; 2 College of Business Administration, American University of the Middle East, Egaila 54200, Kuwait

**Keywords:** auditory modeling, species classification, cochlear filtering, machine learning, sound signal processing

## Abstract

Biomimetic approaches have gained increasing attention in the development of efficient computational models for sound scene analysis. In this paper, we present a sound-based animal species classification method inspired by the auditory processing mechanisms of the human cochlea. The approach employs gammatone filtering to extract features that capture the distinctive characteristics of animal vocalizations. While gammatone filterbanks themselves are well established in auditory signal processing, their systematic application and evaluation for animal vocalization classification represent the main contribution of this work. Four gammatone-based feature representations are explored and used to train and test an artificial neural network for species classification. The method is evaluated on a dataset comprising vocalizations from 13 animal species with 50 vocalizations per specie and 2.76 seconds per vocalization in average. The evaluations are conducted to study the system parameters in different conditions and system architectures. Although the dataset is limited in scale compared to larger public databases, the results highlight the potential of combining biomimetic cochlear filtering with machine learning to perform reliable and robust species classification through sound.

## 1. Introduction

Acoustic communication plays a vital role in the lives of many animal species, serving functions like territory defense, warning signals, and social bonding. This communication has been in the topics of different studies in the last decades [1]. The unique characteristics embedded in animal vocalizations provide a rich source of information about species identity, among others. Leveraging these signatures, sound-based classification methods have been increasingly proposed as non-invasive and efficient at monitoring wildlife populations.

With recent advances in signal processing and machine learning, it has become feasible to automatically classify and identify animals based on their vocalizations [2,3] with high efficiency. Other works have also been using modalities like vision [4]. This capability holds great promise for applications in biodiversity conservation, ecological research, and behavioral studies. For instance, acoustic monitoring can facilitate long-term and large-scale surveys in remote or dense habitats where traditional visual methods are impractical. Despite significant progress, challenges remain. The variability in vocalizations due to environmental noise, individual differences, and contextual factors can complicate the classification process. Furthermore, the development of generalizable models across species does not have a straightforward solution. Model accuracy and loads of computation are also challenging when embedded on real-time systems [2]. Addressing these issues requires a robust understanding of vocal signal features and the implementation of adaptive classification techniques with computationally efficient software architectures. Additionally, difficulties exist in establishing large datasets of selected animal sounds.

Also in the field of audio signal processing, biomimetic approaches rooted in engineering models of the human auditory system have made strong progress. Applications can be found in simulating human cochlear mechanisms with convolutional neural networks [5], finite-element models of the human auditory periphery [6], and wearable biomimetic auditory sensors [7]. Within biomimetic sound signal processing, the research presented in this paper aims to develop and evaluate an approach for classifying animal species through their vocalizations rather than performing detection or identification in the broader sense, thereby focusing on the classification stage of the auditory analysis process. The proposed method combines cochlear-inspired acoustic feature extraction with machine learning algorithms to achieve robust and efficient species classification. Specifically, the approach employs a model of part of the human auditory system—using gammatone filterbanks that mimic the frequency selectivity of the cochlea—to extract biologically meaningful features from animal vocalizations. While gammatone-based feature extraction is an established technique in auditory research, its systematic adaptation and evaluation for animal vocalization classification constitute the core novelty of this study. Specifically, this biomimetic method was chosen because it models the frequency selectivity and nonlinear characteristics of the human cochlea, providing a perceptually meaningful representation of sound. Such a representation captures subtle spectral cues present in animal vocalizations. The extracted features are then classified using a lightweight two-layer neural network. The study explores a dataset containing vocalizations from diverse species and contributes to the expanding field of bioacoustics by supporting scalable, automated methods for wildlife monitoring and conservation. By emphasizing biological plausibility, computational efficiency, and effective classification performance, this work offers a novel biomimetic perspective to species classification in bioacoustics, illustrating the potential of nature-inspired signal processing in advancing automated ecological monitoring technologies.

The rest of the paper is organized as follows. Section 2 shows different work done in the literature of animal classification through sound and related fields. Section 3 shows the design of the proposed approach in detail, from exploitable and extracted features to the employed machine learning framework. Section 4 shows the different evaluations made on the approach and their results. Section 5 shows a discussion of the proposed approach and the obtained findings, and Section 6 concludes the paper and expands to future work.

## 2. Related Work

The swift advancement of machine learning and deep learning has significantly impacted the field of acoustic species classification, especially in the classification of animal and environmental sounds. Convolutional neural networks (CNNs), recurrent neural networks (RNNs), transformers, and pretrained audio models are some of the methods that have gained popularity recently. In their 2022 study, Sun et al. focused on bioacoustic classification in a hyperdiverse rainforest and demonstrated how CNNs in conjunction with data augmentation techniques could handle complex natural sounds. They were able to identify over 30 species in extremely noisy real-world datasets with high accuracy [8]. DualDiscWaveGAN, a novel GAN-based data augmentation technique, was presented in [9] and enhanced the classification robustness for rare and unbalanced animal sound classes. Additional noteworthy contributions were made in [10], where deep learning was used to detect forest species and implemented custom models in real-time conservation applications; Nolasco et al. [11], who showed that few-shot learning techniques could achieve accurate detection with as few as five samples per species, allowing for low-resource applications; and Sisodia et al. [12], who suggested a composite deep learning model with augmented features that increased accuracy across multiple animal datasets.

High-dimensional audio data was handled utilizing hybrid frameworks that included feature extraction and deep classifiers in recent developments by Yang et al. [13], which focused on feature selection and optimization in animal sound classification pipelines. Studies like Adami et al. [14] went beyond categorization to concentrate on applications like intelligent animal repelling systems, which combine acoustic monitoring and embedded AI to provide real-time agricultural intervention [14]. Similar to this, Cheng et al. [15] suggested edge–cloud task offloading techniques for mobile networks’ audio recognition, which might be applied to rural areas’ animal detection situations, while Lin et al. [16] demonstrated a specialized wild animal detection system for biodiversity monitoring [16]. In order to connect sound event recognition with real-world implementation, Sharanyaa et al. [17] developed a machine learning pipeline for animal and bird detection. Zhang [18] suggested a straightforward yet powerful sound-based recognition model that can be modified for edge deployment in conservation settings, while Xu et al. [19]) created a multi-view CNN system to enhance species recognition across overlapping acoustic environments. The performances of several previous studies are reported in Table 1.

While numerous studies have employed features such as Mel-Frequency Cepstral Coefficients (MFCCs) and deep convolutional neural networks (CNNs) for animal sound classification, our approach introduces a biomimetic and computationally lightweight alternative. The use of gammatone filtering, which approximates the human auditory filterbank response, has been successfully applied in various sound processing tasks [20,21]. Gammatone filters offer the potential to capture spectral cues that are better aligned with natural auditory perception and may outperform conventional features when distinguishing between a wide variety of animal vocalizations. In contrast to many existing approaches that focus on closely related species or require large, computationally intensive models, the proposed method is evaluated across 13 species, demonstrating strong generalization without reliance on complex architectures. The selected two-layer neural network provides greater adaptability to complex data compared to traditional classifiers such as K-nearest neighbors, ensemble classifiers [22], Gaussian Mixture Models [23], and similarity-based pattern matching [24]. Additionally, in comparison with more sophisticated classifiers like convolutional and deep learning architectures, the proposed system achieves competitive accuracy with lower computational requirements and improved interpretability.

## 3. Proposed Design

### 3.1. Methodology

The proposed animal species classification approach leverages biologically inspired cochlear filtering features to extract rich and representative vocalization characteristics. The method consists of the following major steps:

- Cochlear Filtering: Audio signals undergo a cochlear filterbank modeled after human auditory filtering, which decomposes the signal into frequency bands reflecting cochlear frequency selectivity. This approach captures fine-grained spectral and temporal patterns relevant to animal vocalizations.
- Feature Extraction: Several features are extracted, including time–frequency information, designed to highlight distinguishing vocal characteristics across species, along with cochlear filtering-based features. These features aim to emulate auditory perception more closely than traditional acoustic features.
- Classification Using Neural Networks: The extracted features are fed into neural network architectures trained to classify animal species. We experiment with feed-forward neural networks to capture vocalization patterns.
- Evaluation: The method is evaluated using a dataset consisting of vocalizations from 13 animal species. Performance evaluation consisted of confusion analysis along with the following metrics:
  − Accuracy: Calculated as the ratio of correct predictions to the total number of tests, across all species.
  − Precision: Calculated for each class as the ratio of predictions correctly classified as belonging to the class, to the total number of predictions classified as belonging to the class. The average precision over all the classes is reported in the paper.
  − Recall: Calculated for each class as the ratio of correct predictions from the class, to the total number of tests from the class. The average recall over all the classes is reported in the paper.
  − F1-score: Calculated for each class *i* according to the following formula:
    (1) $$F1_{i}=\frac{2\times P_{i}\times R_{i}}{P_{i}+R_{i}}$$
    where $P_{i}$ and $R_{i}$ are, respectively, the calculated precision and recall for the class *i*. The average F1-score over all the classes is reported in the paper.

### 3.2. Species Sound Features

**Figure 1:**
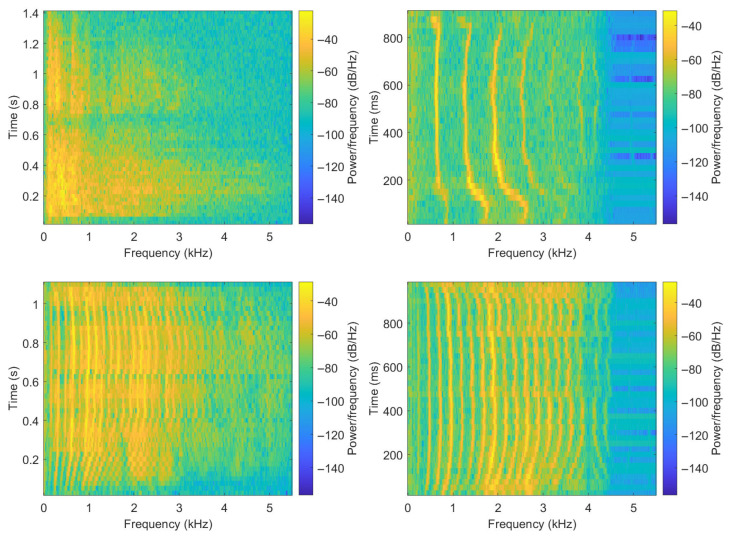
Spectrograms of sample vocalizations from different animal species. **Top-left**: lion, **top-right**: cat, **bottom-left**: cow, **bottom-right**: sheep.

Before showing the extracted features, a visualization and analysis of vocalizations from different species are performed in this part. Figure 1 shows four spectrograms obtained based on signals sampled at 11,025 Hz with frames of 50 ms, and an overlap of 50% between consecutive frames. From the figure, the following statements can be made. Some vocalizations can be seen as sequences of more or less active sounds, with the time duration and spacing of highly active sounds being species-dependent. Also, animals do not repeat the same sound, and vocalizations can consist of different sounds combined. Additionally, the ranges of frequencies with relevant sound activities are species-dependent. Finally, in some cases more than others, the vocalizations show sequences of active frequencies like fundamentals and harmonics.

### 3.3. Feature Extraction

As shown previously, different characteristics of the animal vocalizations can be used as discriminant features, allowing a classification algorithm to learn the corresponding species. In this work, the different features extracted from the animal vocalizations are the following.

#### 3.3.1. Vocalization Duration

For the vocalization *i* of the species *s*, the duration $d_{s,i}$ is taken as one of the discriminant features.

#### 3.3.2. Rate of Active Frames

Over the vocalization, and regardless of frequency content, some frames may involve more activity than others. The rate of frames with energies exceeding a certain threshold is considered among the discriminant features used. For the vocalization *i* of the species *s*, this rate $R_{s,i}$ is calculated as follows: considering that the vocalization consists of $N_{s,i}$ frames of NS samples each, the Short-Time Fourier Transform (STFT) $ft_{s,i,n}$ is calculated for each frame, and it is not clear if those spectra also use the overlapping of frames explained in Section 3.2. Indeed, it would have been more coherent with the rest of the paper to also use gammatone filters in this computation to obtain a biomimetic energy, but apparently this is not the case. This is also applicable to the computation of active frequencies (Section 3.3.3). *n* and their energies are estimated as

(2) $$E_{s,i,n}=\sum_{j=1}^{NF}{\left|ft_{s,i,n}\left(j\right)\right|}^{2},$$

where $NF=\frac{NS}{2}$. The energy calculation is performed in the Fourier domain as this provides a convenient and efficient means of identifying the interval of active frequencies in each vocalization sample. This spectral representation allows us to determine the dominant frequency bands and energy distribution. The maximum and minimum frame energies over the considered vocalizations are approximated as $ME_{s,i}=\max_{n=1}^{N_{s,i}}E_{s,i,n}$ and $mE_{s,i}=\min_{n=1}^{N_{s,i}}E_{s,i,n}$. Then, the energy threshold is calculated as

(3) $$TE_{s,i}=mE_{s,i}+E_{T}\times \left(ME_{s,i}-mE_{s,i}\right),$$

where $E_{T}$ is a parameter that can be used to adjust the selectivity of this process. Then, the number $HE_{s,i}$ of frames satisfying the condition $E_{s,i,n}>TE_{s,i},n=1\ldots N_{s,i}$ is calculated and the rate $R_{s,i}$ is calculated as

(4) $$R_{s,i}=\frac{HE_{s,i}}{N_{s,i}}.$$

#### 3.3.3. Interval of Active Frequencies

The interval of active frequencies is defined by the lowest and highest frequencies where the activity exceeds a certain threshold. This process is performed as follows. The STFT $ft_{s,i,n}$ consists of NF points showing the signal activities $|ft_{s,i,n}\left(j\right)|,j=1\ldots NS/2$ at frequencies ranging up to $\frac{f_{s}}{2}$ where $f_{s}$ is the signal sampling frequency. The minimum and maximum activities are named as $mft_{s,i}$ and $Mft_{s,i}$, respectively. In a similar way with the energy thresholding process, a threshold is calculated for the spectral activity as

(5) $$Tft_{s,i}=mft_{s,i}+ft_{T}\times \left(Mft_{s,i}-mft_{s,i}\right),$$

where $ft_{T}$ is an adjustable parameter. All the frequencies satisfying the condition $|ft_{s,i,n}\left(j\right)|>Tft_{s,i},j=1\ldots NS/2$ are then found, and their minimum and maximum values $Lf_{s,i}$ and $Hf_{s,i}$ are used as features.

#### 3.3.4. Cochlear Filtering Features

**Figure 2:**
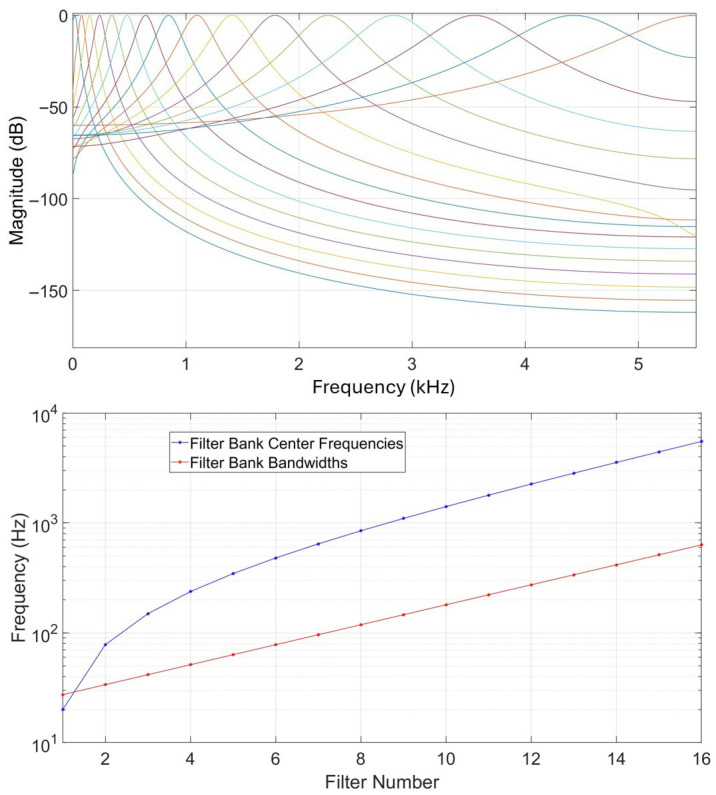
Illustration of a bank of 16 gammatone filters. **Up**: magnitude responses in dB. Each colored line corresponds to a specific filter and colors can be the same by coincidence. **Down**: filter center frequencies and bandwidths.

Cochlear filtering is an important part of human hearing, taking place in the inner ear. Gammatone filters have been implemented as a computational model for human cochlear filtering [25]. A gammatone filterbank consists of filters regularly spaced on the equivalent rectangular bandwidth as their center frequencies are distributed in proportion to their bandwidth. Figure 2 illustrates a filterbank of 16 filters based on a sampling frequency of 11,025 Hz, showing their frequency responses, center frequencies, and bandwidths. Acoustic features relying on gammatone filtering have been previously used in different works addressing human speaker recognition [26,27,28] and sound source localization [20].

In this work, cochlear features are extracted from the signals over short-time frames with 50% overlap between consecutive frames as follows.

1. Filter the signal with the bank of *N* gammatone filters to obtain *N* output signals.
2. Compute the *N* output energies S[g] (g=1…N) of the *N* filters.
3. Compute the discrete cosine transform (DCT) of the energy logarithms. This gives the vector of *N* coefficients $GC_{s,i,n}=DCT\left(log_{10}\left(S\left[g\right]\right)\right)$, g=1…N for each frame.

Thus, for each recording, $N_{s,i}$ vectors are obtained. Additionally, each vector is combined with its corresponding Delta vector: the difference between it and the previous vector. This allows us to track the evolution of the dynamics in the sound content. But to represent the entire recording, their average and standard deviations, $AVGC_{s,i}$ and $STDGC_{s,i}$, respectively, can be calculated and used as features.

#### 3.3.5. Feature Vector Formation

**Figure 3:**
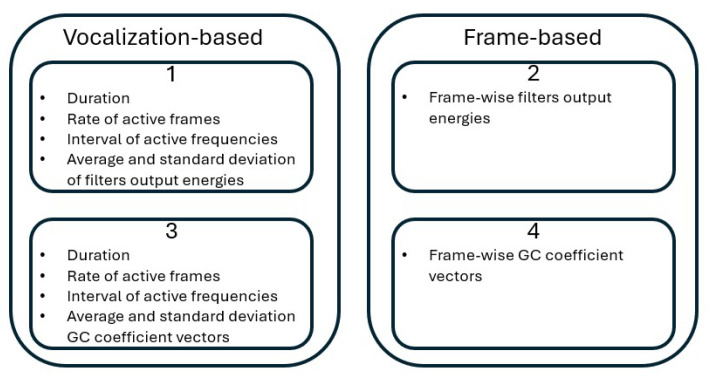
A block diagram illustrating the feature computation possibilities used.

After calculating the different features, feature vectors used for training and testing the approach are extracted in four possibilities shown below and illustrated in Figure 3. Possibilities 3 and 4 use all the feature extraction steps of the cochlear filtering (all three steps shown in Section 3.3.4), while possibilities 1 and 2 use the output energies of the gammatone filters without the logarithm and DCT computations (first two steps shown in Section 3.3.4 only). Thus, possibilities 1 and 2 precede possibilities 3 and 4 in their computational steps.

1. A feature vector $V_{s,i}$ is obtained for each species’ vocalization as follows:
  (6) $$V_{s,i}=\left[d_{s,i},R_{s,i},Lf_{s,i},Hf_{s,i},AVGC_{s,i},STDGC_{s,i}\right].$$
  But in this case, GC vectors are obtained as the filters’ output energies only without the logarithm and DCT calculation shown in the third point in Section 3.3.4.
2. A feature vector is obtained for each short time frame and is composed solely of the $GC_{s,i,n}$ feature vector. But also in this case, GC vectors are obtained as the filters’ output energies only without the logarithm and DCT calculation shown in the third point in Section 3.3.4.
3. A feature vector $V_{s,i}$ is obtained for each species’ vocalization as
  (7) $$V_{s,i}=\left[d_{s,i},R_{s,i},Lf_{s,i},Hf_{s,i},AVGC_{s,i},STDGC_{s,i}\right].$$
4. A feature vector is obtained for each short time frame and is composed solely of the $GC_{s,i,n}$ feature vector.

It can be seen that fewer feature vectors are obtained from the first and third possibilities, but more information covering the other properties of the vocalization is available. All possibilities will be used and evaluated in Section 4.

### 3.4. Neural Network

As a classifier using the vectors $V_{s,i}$ as inputs, a neural network is used in this study. The network has the characteristics shown in Table 2. The network was limited to a maximum number of training iterations of 1000, but required less to converge in many cases. Additionally, training was stopped when the loss or its gradient fell below $1\times 10^{-6}$.

## 4. Testing and Evaluation

### 4.1. Used Database

To evaluate the proposed approach, an online available database [30] was used. This database contains sound recordings of vocalizations from 13 species with 50 vocalizations per specie. The species are lions, bears, cats, chickens, cows, dogs, dolphins, donkeys, elephants, frogs, horses, monkeys, and sheep. The recordings were sampled at different sampling frequencies, but to ensure consistency and comparability across all samples, all signals were resampled to 11,025 Hz, which is common to many of the existing recordings, aside from the fact that it is sufficiently high to capture relevant spectral content of animal vocalizations while keeping computational costs low for large-scale processing. The signals were also high-pass filtered with a frequency of 20 Hz to remove very low-frequency components such as microphone rumble, wind noise, or handling noise, which do not carry species-specific information and can negatively affect the subsequent feature extraction. This preprocessing step ensured that all signals entered the feature extraction pipeline with a uniform bandwidth and without low-frequency artifacts, thereby improving the stability and reliability of the analysis. In the raw data, the vocalization durations were characterized by the values shown in Table 3. While the dataset is limited in scope, it provides a platform to demonstrate the viability of cochlear-based features in species classification. Future expansions will include larger and more diverse datasets.

### 4.2. Tests

Multiple tests have been made with the different options of feature vector formation shown in Section 3.3.5 and the presented database. To reduce the effect of the available recordings conditions on the performances, a 5-fold cross-validation is performed by shuffling the recordings five times and each time, the first 45 were used to make the training data and the remaining 5 were used for testing (90% training, 10% testing). In this implementation, and in constrast with standard 5-fold procedures, the fixed 90–10 split was repeated five times with different random partitions, which effectively preserves the idea of multiple validation cycles while ensuring a larger training set given the limited dataset size. This approach was chosen to maximize the amount of data used for model learning in each run while still assessing generalization across multiple random splits. Additionally, the tests have been conducted with the following parameters: N=30, $E_{T}=0.005$, and $ft_{T}=0.1$.

#### 4.2.1. Filter Output Energies

Table 4 shows the results of five different tests made according to the points 1 and 2 in Section 3.3.5. For frame-base tests, results are based on a frame duration of 40 ms and an energy-based silence removal process. Also in this case, the number of training samples per class has been fixed to be the same for all classes. This number is the lowest number of training frames among all the classes. A certain deviation between the results of five different tests can be seen, which can be explained mainly by the usage of different training and testing data each time. Also, the vocalization-based accuracy is higher than the frame-based accuracy in all tests; however, it does not offer a significant advantage.

#### 4.2.2. Filter Output Energies with Logarithm and DCT

Table 5 shows the results obtained with features extracted based on points 3 and 4 in Section 3.3.5. Also in this case, frame-based tests use frames of 40 ms, silence removal and employ the same number of training samples for all classes. By comparing Table 4 and Table 5, an improvement due to adding the logarithm and DCT can be noted. Also, a higher accuracy can be noted with vocalization-based features over frame-based features. However, this advantage cannot be solidly built upon due to the limitation of the recordings in the used database which limits both numbers of training and testing samples. Additionally, the frame-based results allow us to perform more operations that can improve the overall approach performance as it will be shown in the coming paragraphs.

**Figure 4:**
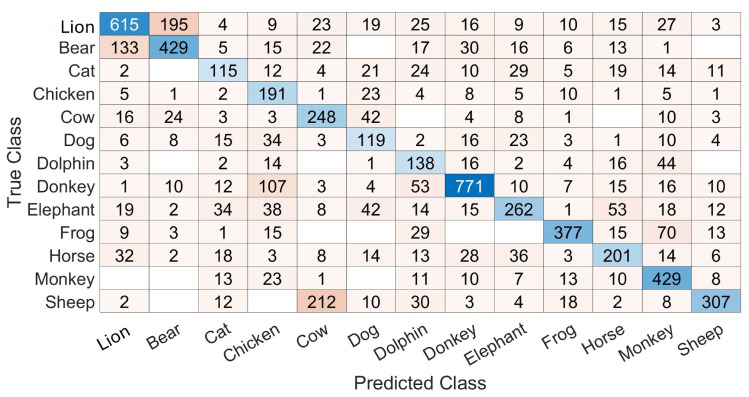
A sample confusion matrix with the training and testing based on point 2 in Section 3.3.5. The results are on a frame-by-frame basis. The cell color is darker when the number inside the cell insreases and the diagonal is colored in shades of blue.

Figure 4 shows a sample confusion matrix obtained with training and testing on a frame-by-frame basis. This matrix is filled with numbers of frames in each cell and allows us to see the differences between the numbers of frames available for testing in each class. Also, it allows us to see different confusions and a variability in recognitions. For example, the recognition rate is 63.4% for the lion while it is 62.45%, 43.23%, 74.23% and 50.49% for the bear, cat, chicken and sheep, respectively.

#### 4.2.3. Tests Based on Longer Durations

**Figure 5:**
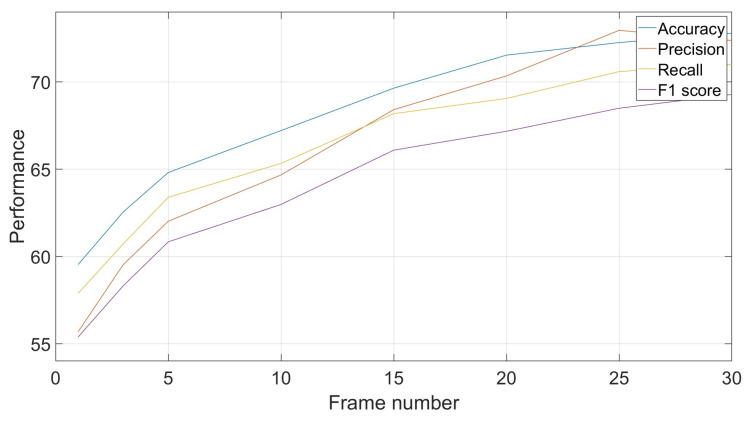
Effect of frame number on classification performances: accuracy, precision, recall and F1-score.

As noted in Section 4.2.2, frame-based training and testing allows for more flexibility in exploiting classification results in a way to improve the performances. This can be done by exploiting classifications of a certain number of consecutive frames, which can be different, to obtain a unique group-based decision through the mode of these classifications. For example, if in a sequence of five short-duration classifications, the results are [*class1*, *class1*, *class3*, *class1*, *class5*], the common decision is *class1* by majority voting. Figure 5 shows the evolutions of results based on different numbers of frames. It shows an improvement in performances with increased number of frames, with a tendency to converge to a certain recognition rate. In the shown example, the recognition rate increased by around 14% when moving from a frame-by-frame decision to a decision based on groups of 25 frames each.

#### 4.2.4. Frame Duration Effect

**Figure 6:**
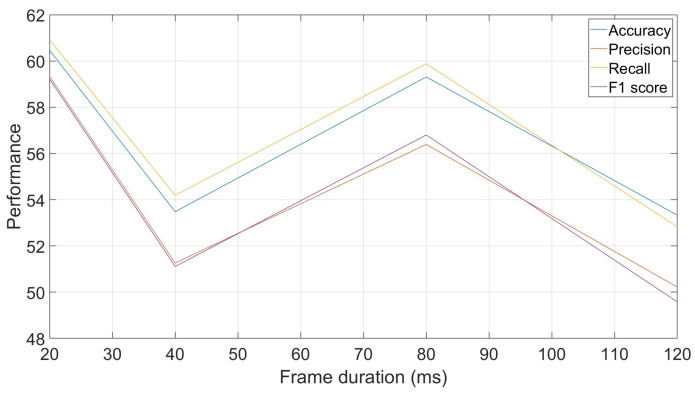
Effect of frame duration on classification performances: accuracy, precision, recall and F1-score.

Another parameter that can be exploited when using frame-by-frame decisions is the frame duration used. The reported results so far have been based on frames of 40 ms each. However, this duration, which can be used in human voice-based tasks, can be adjusted to explore faster or slower evolutions of the signals in the animal vocalizations. Figure 6 shows performances obtained with different frame durations, ranging from 20 ms to 120 ms. The curve allows us to see that there is no clear trend in the evolution of the recognition rates with the evolution of the frame duration, as the recognition rate at 40 ms is lower than its two surrounding durations, 80 ms and 20 ms.

**Figure 7:**
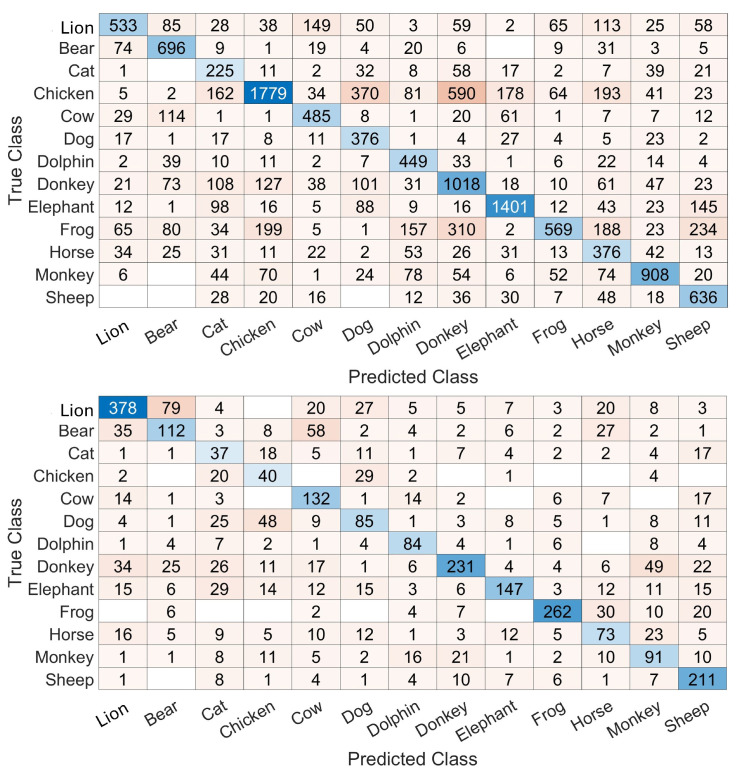
**Top**: confusion matrix based on 20 ms frames. **Bottom**: confusion matrix based on 80 ms frames. The results are on a frame-by-frame basis. The cell color is darker when the number inside the cell insreases and the diagonal is colored in shades of blue.

Confusion matrices obtained with the 20 ms and 80 ms durations are shown in Figure 7. As expected, the number of frames is higher with 20 ms frames. But another result, which is less intuitive, is the inter-class variability of performances due to the change in frame durations. For example, for the classs frog and chicken, the recognition rates evolve from 30.48% and 50.51% to 76.83% and 40,82%, respectively, when frame durations evolve from 20 ms to 80 ms, respectively. Additionally, the patterns of confusions are different for certain classes with different frame durations. This can be seen in the classes sheep, lion, and bear, for example. These indications could result in concluding with a frame duration that can fit one class more than other classes, or more than other frame durations. Such a fact can be exploited to improve the performances.

### 4.3. Neural Network Training

**Figure 8:**
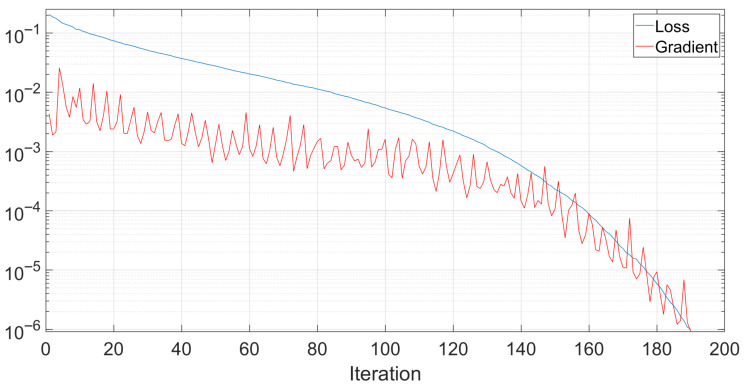
An example of the evolution of the neural network loss and gradient during training.

Throughout the performed tests, the training data of the neural network was observed to investigate its speed and effectiveness. Figure 8 shows the evolution of the training loss with respect to the training iteration number. As shown, the training stopped after 190 iterations. In the shown example, the training lasted for around 2.2 s on a PC running Microsoft Windows 11 on an Intel Core i7-12650 processor with 32 GB of RAM and an NVIDIA GeForce RTX 4060 graphical processing unit.

### 4.4. Parameter Effects

Among the important parameters used in the design of this study, $E_{T}$ and $ft_{T}$ are of high importance. Indeed, lowering $E_{T}$ may lead to allowing frames with insignificant sound activity to be used in the training and testing processes, affecting the neural network training and testing. Also, increasing $E_{T}$ leads to discarding frames with potential relevant specie discriminatory information. Similarly, increasing and decreasing $ft_{T}$ can lead to considering frequencies with insignificant activity or discarding significant frequencies. A study has been performed with different values of $E_{T}$ and $ft_{T}$ in order to study their effects more closely, and the results are reported in Table 6 and Table 7. As shown, increasing $E_{T}$ above 0.005 reduces the percentage of kept frames to less than 74% and increasing $ft_{T}$ above 0.5 reduces the frequency interval considered as containing significant activity to less than 56% of the possible interval.

### 4.5. Adjusted Approach

**Figure 9:**
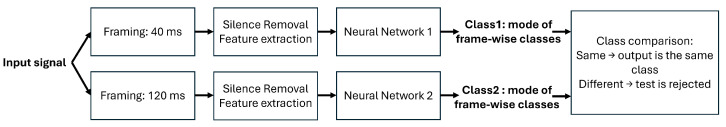
Proposed architecture for a dual neural network approach.

Based on the results obtained in the previous parts, a new architecture is proposed. This architecture relies on two neural networks trained with features extracted based on two different time frame durations. Figure 9 shows the architecture and depicts the classification decision, which is done when the two neural networks output the same class. It should be noted that each neural network decision is a unique class label based on the decisions of all the input frames, being the mode of the frame-by-frame decisions.

In one of the tests, this approach produced a recognition rate, not taking into account rejected tests, of 91.49%, with most classes being recognized with a 100% accuracy. In this sample test, around 12% of the tests were rejected and the recognition rate is based on the remaining ones. Like in the previous evaluations, different trials involving different neural network initializations and different training and testing data lead to different recognition rates. However, the deviations between different trials are not significant.

## 5. Discussion

The reported results demonstrate the effectiveness of a cochlear filtering modeling-based feature extraction combined with a machine learning approach in identifying animals through sounds of their vocalizations. Different aspects of the proposed approach can be discussed as follows and as summarized in Table 8.

Over the different results reported in Table 4 and Table 5, it was seen that vocalization-based accuracies were higher than frame-based accuracies. This can be explained by the fact that more information is included in full vocalizations than in individual short time frames. However, other features than average and standard deviation can be exploited in future work to reflect the information present in the vocalizations. Additionally, more variability in vocalization-based accuracy than in frame-based accuracy has been seen. This is due to the fact that vocalizations are more diverse with different durations and content. It was considered that each recording in the database corresponds to a vocalization. Thus, when shuffling the vocalizations for training and testing over different trials, results were more prone to variations.

In the literature of sound-based biodiversity studies, Mel-Frequency Cepstral Coefficients have been widely used [31,32,33]. When used in the current framework instead of the gammatone-based features, MFCCs provide a frame-by-frame accuracy revolving around 49% in different trials with 16 coefficients, underperforming all the reported results in Table 5. This shows the advantage provided by the gammatone filterbank-based feature extraction compared to the triangular filterbank-based MFCCs in the studied task.

**Figure 10:**
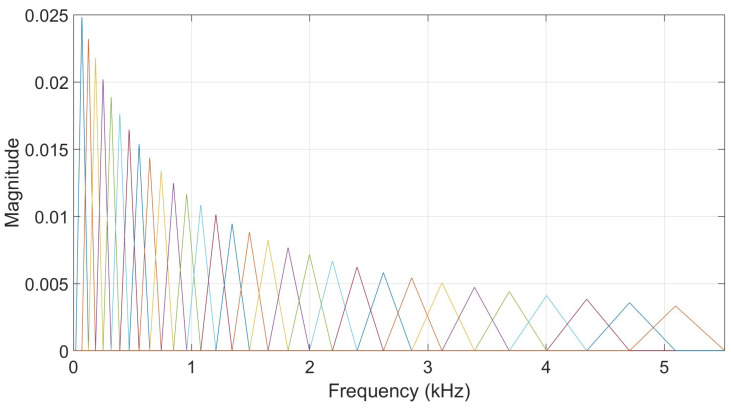
An illustration of a triangular filterbank. Each colored line corresponds to a specific filter and colors can be the same by coincidence.

However, to compare the two types of filterbanks directly, a comparison has been done between the results based on a neural network trained with the output energies of the gammatone filterbank and another one trained with the output energies of a triangular filterbank like the one illustrated in Figure 10. This filterbank consists of filters regularly spaced on the mel-scale and with the same number of filters used in the gammatone filterbank as well as the same frequency interval. Two tests were performed with the same samples that were used in both filtering approaches to ensure that that comparison is done in the same conditions. The frame duration used was 20 ms. The results are reported in Table 9. These results suggest that both filterbanks are capable of producing competitive outcomes, depending on the characteristics of the data and the experimental setup. Nevertheless, the gammatone filterbank offers an additional advantage in its biomimetic design, as it more accurately models the auditory filtering process of the human cochlea. This physiological grounding provides a stronger conceptual foundation and may lead to better generalization across diverse animal vocalization datasets and acoustic environments.

Also in the literature, deep machine learning models like convolutional neural networks [33,34] and recurrent neural network variants [32] have been proposed. Compared to such models, and despite the high performances that they can offer, the neural network used in this study shows different benefits. Indeed, the proposed two-layered network requires lower computational costs, can train faster, requires less data, and can be easily adjusted and deployed for real-time operation on a limited-resource portable computer, for example.

Another potential benefit of the neural network architecture is its suitability for handling data variability and uneven sample distributions, which are common in real-world bioacoustic datasets. The database used in this study consists of vocalizations recorded under non-controlled conditions, resulting in differences in duration and quality among species. While this work does not explicitly evaluate performance under data imbalance, the results indicate that the proposed approach remains stable across species despite these variations.

Although gammatone filterbanks have been widely used in auditory and speech processing, their systematic adaptation and evaluation for animal vocalization classification remain limited in the literature. The present study bridges this gap by applying biomimetic cochlear filtering to the field of bioacoustics and analyzing its comparative performance against classical triangular filterbanks. The results demonstrate that while both approaches yield competitive outcomes—with each showing slight advantages under different test conditions—the gammatone filterbank offers a biologically meaningful representation that aligns with the frequency selectivity of the human cochlea. This alignment not only provides interpretability grounded in auditory physiology but may also enhance generalization to diverse animal species and recording conditions. Hence, the contribution of this work lies in contextualizing an established auditory model within a novel application domain, supported by empirical validation and comparative analysis.

## 6. Conclusions

This paper presents a novel biomimetic sound-based animal species classification approach that integrates auditory-inspired feature extraction with a machine learning classification framework. By drawing inspiration from the human cochlea through the use of gammatone filtering, the proposed method captures biologically relevant acoustic features that enable accurate species classification. Experimental results demonstrate that the approach achieves strong classification performance, with results influenced by both the nature of the extracted features and the feature extraction process itself. Importantly, the method proves effective even when handling limited and variably recorded vocalization data across multiple species.

Overall, this work demonstrates the potential of biomimetic auditory processing in advancing bioacoustic species classification, offering a promising avenue for enhancing ecological monitoring through the lens of nature-inspired computation.

### Future Work

Future work will focus on extending this study using larger and more diverse animal vocalization datasets to further validate generalization across species and environments. Additional research will explore alternative cochlear-inspired models, such as adaptive gammatone filters or neural auditory front-ends, to enhance temporal and spectral resolution. Combining biomimetic and conventional spectral features will also be investigated to improve robustness against noise and signal variability. Finally, efforts will be directed toward real-time implementation and deployment on embedded systems for automated wildlife monitoring applications.

## Figures and Tables

**Table 1:** Performance summary of recent studies on animal sound classification (2022–2024).

Ref.	Approach/Model	Dataset/Domain	Reported Performance
[8]	Sun et al. (2022)	CNN with data augmentation	Rainforest animal sounds (Ecological Indicators); Accuracy: 94.1%, F1-score: 93.5%
[9]	Kim et al. (2023)	DualDiscWaveGAN + CNN classifier	Custom animal sound dataset (Sensors); Accuracy: 95.3%, Precision: 94.8%
[10]	Mohmmad et al. (2023)	Deep CNN for wild animal detection	Forest environment recordings (ICSES 2023); Accuracy: 89.7%
[11]	Nolasco et al. (2023)	Few-shot learning (5-example detection)	EcoInformatics dataset; Accuracy: 85.0%, F1-score: 82.3%
[12]	Sisodia et al. (2024)	Composite deep learning (CNN + LSTM + feature fusion)	CoDIT 2024 dataset; Accuracy: 96.2%, F1-score: 95.8%
[13]	Yang et al. (2024)	Optimized CNN framework	Public bioacoustic dataset (arXiv:2407.03440); Accuracy: 97.1%, Recall: 96.3%
[16]	Lin et al. (2023)	AI-based wild animal detection system	Real-time camera trap and acoustic data; Accuracy: 90.5%

**Table 2:** Used neural network characteristics.

Characteristic	How Applied in This Work
Number of inputs	dimension of $V_{s,i}$
Number of hidden layers and cells	2 hidden layers, 50 and 25 cells, respectively
Hidden cell activation function	rectified linear unit (ReLU)
Output cells activation function	softmax
Solver	Broyden–Fletcher–Goldfarb–Shanno quasi-Newton algorithm [29]

**Table 3:** Average, standard deviation, maximum, and minimum vocalization durations among the 50 recordings available in the database for each of the 15 species. All the values are expressed in seconds.

Specie	Average	Standard Deviation	Maximum	Minimum
Lion	4.55	3.20	13.86	0.91
Bear	2.30	1.02	5.16	0.98
Cat	1.45	0.72	3.95	0.5
Chicken	4	8.70	54.29	0.26
Cow	1.97	0.87	4.65	0.77
Dog	1.58	0.81	4.03	0.27
Dolphin	1.84	0.93	7.15	0.78
Donkey	4.21	4.14	18.5	0.76
Elephant	2.97	1.54	7.23	0.91
Frog	2.71	2.54	9.94	0.22
Horse	1.83	0.89	3.84	0.32
Monkey	4.19	2.63	9.93	0.2
Sheep	2.37	2.1	8.71	0.46

**Table 4:** Accuracies, average class precisions, recalls and F1 scores, calculated in percent, calculated in percent, with features extracted based on points 1 and 2 in Section 3.3.5.

Trial	Frame-Based				Vocalization-Based			
	Accuracy	Precision	Recall	F1 Score	Accuracy	Precision	Recall	F1 Score
1	51.79	49.28	51.55	48.62	70.31	73.37	69.61	69.07
2	50.01	49.77	50.62	48.66	70.31	74.80	70.38	69.21
3	49.81	47.26	48.75	46.62	71.87	74.16	71.53	69.92
4	50.86	47.67	50.66	47.28	75.00	78.17	75.00	75.32
5	47.26	45.80	47.21	44.88	73.43	77.07	73.84	71.50
Average	49.71	47.96	49.76	47.21	72.18	75.51	72.07	71.00

**Table 5:** Accuracies, average class precisions, recalls and F1 scores, calculated in percent, with features extracted based on points 3 and 4 in Section 3.3.5.

Trial	Frame-Based				Vocalization-Based			
	Accuracy	Precision	Recall	F1 Score	Accuracy	Precision	Recall	F1 Score
1	58.21	54.96	58.18	55.19	71.87	74.14	71.54	71.98
2	58.39	55.72	55.91	54.84	68.75	72.16	68.84	69.07
3	66.22	63.66	63.57	62.71	78.12	80.57	78.46	77.94
4	58.71	54.93	56.70	54.58	68.57	73.88	68.84	67.85
5	60.46	59.33	60.92	59.22	71.87	74.49	71.53	71.62
Average	60.40	57.72	59.06	57.31	71.84	75.05	71.84	71.69

**Table 6:** Effect of $E_{T}$ on the percentage of frames kept for training and testing.

$E_{T}$	0.0001	0.0005	0.001	0.005	0.01
Percentage of kept frames	91.26	85.28	82.02	73.65	69.04

**Table 7:** Effect of $ft_{T}$ on the reduction in the considered frequency interval.

${\mathrm{ft}}_{T}$	0.01	0.05	0.1	0.5
Considered frequency interval over possible interval	97.8	92.52	84.39	55.44

**Table 8:** Summary of discussion points on the effectiveness of cochlear filtering modeling-based feature extraction and machine learning for animal vocalization recognition.

Aspect	Observation/Result	Explanation/Interpretation	Implications/Future Work	Reference(s)
Vocalization vs. Frame Accuracy	Vocalization-based accuracies higher than frame-based accuracies; more variability in vocalization accuracy.	Full vocalizations contain more information than short frames; variations due to diverse durations and content.	Future work could explore additional features beyond average and standard deviation to better capture vocalization information.	Current study results
Comparison with MFCCs	MFCCs achieved ∼49% frame-level accuracy, lower than gammatone-based features.	Gammatone filterbank better models auditory processing than MFCC’s triangular filterbank.	Confirms the advantage of cochlear-inspired features for biodiversity tasks.	[31,32,33]
Comparison with Deep Learning Models	CNNs and RNNs have been proposed and perform well, but the two-layer network used here shows practical benefits.	The proposed NN is simpler, requires lower computational resources, trains faster, works with less data, and can run in real time on limited hardware.	Enhances suitability for field biodiversity monitoring and portable systems.	[32,33,34]

**Table 9:** Accuracies, average class precisions, recalls and F1 scores, calculated in percent, with features extracted based on point 2 in Section 3.3.5.

Trial	Gammatone Filtering-Based				Triangular Filtering-Based			
	Accuracy	Precision	Recall	F1 Score	Accuracy	Precision	Recall	F1 Score
1	46.49	44.96	48.06	44.56	45.72	44.85	47.56	44.20
2	52.23	49.50	50.12	47.70	53.48	50.77	51.55	49.48

## Data Availability

The original contributions presented in this study are explained in the article. Further inquiries can be directed to the corresponding author.
